# Askin's Tumour: A Report of a Rare Case

**DOI:** 10.7759/cureus.63345

**Published:** 2024-06-28

**Authors:** Prajakta Ghewade, Samarth Shukla, Sunita Vagha, Babaji Ghewade, Pravin Gadkari

**Affiliations:** 1 Department of Pathology, Jawaharlal Nehru Medical College, Datta Meghe Institute of Medical Sciences, Wardha, IND; 2 Department of Respiratory Medicine, Jawaharlal Nehru Medical College, Datta Meghe Institute of Medical Sciences, Wardha, IND

**Keywords:** ewing’s sarcoma, thoracopulmonary region, peripheral pnets, pnets, askin tumour

## Abstract

Primitive neuroectodermal tumours (PNETs) are rare, malignant tumours arising from primitive nerve cells. PNET of the chest wall is rare and is observed in children and young adults. Askin defined Askin's tumour as a PNET of the thoracopulmonary area. It develops from the soft tissues of the chest wall, particularly in the paravertebral region. Here, we report a case of Askin's tumour, a rare neoplasm occurring in the thoracopulmonary region in a 13-year-old girl. She came with complaints of fever, cold, cough with mucoid expectoration, breathlessness for 15 days, and generalized weakness for three months. A high-resolution computed tomography (HRCT) thorax scan was also done, which suggested a large right pleural-based mass with vertebral metastasis. Through diligent diagnostic evaluation involving imaging studies and histopathological examination, the tumour was accurately identified.

## Introduction

Rare malignant tumours called primitive neuroectodermal tumours (PNETs) originate from primitive nerve cells. These are malignant, small, round-cell tumours that are very aggressive. They are frequently referred to as CNS (central nervous system) or central PNETs and can manifest as medulloblastomas in the CNS. Peripheral PNETs are those that appear outside the CNS, that is in the peripheral nervous system. Peripheral PNETs may manifest in the extremities, paravertebral region, pelvis, or chest wall. Usually, these tumours appear in early adulthood or childhood. They may, nevertheless, also manifest at other ages. Here, we present a case of a 13-year-old girl who has Askin's tumour, a rare neoplasm that occurs in the thoracopulmonary region.

## Case presentation

**Figure 1:**
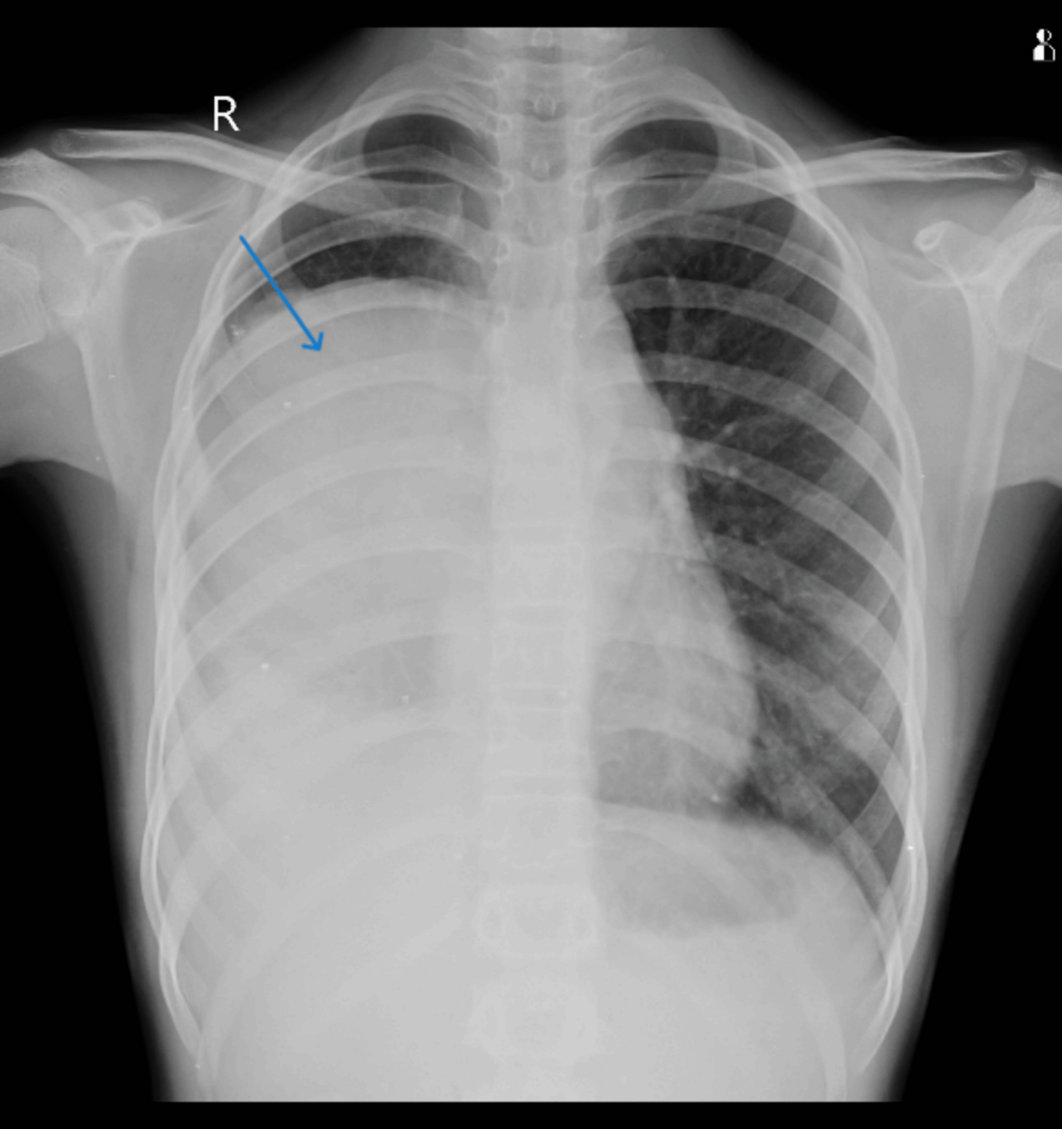
Chest radiograph PA view showing a large homogeneous opacity (blue arrow) PA, posteroanterior view.

**Figure 2:**
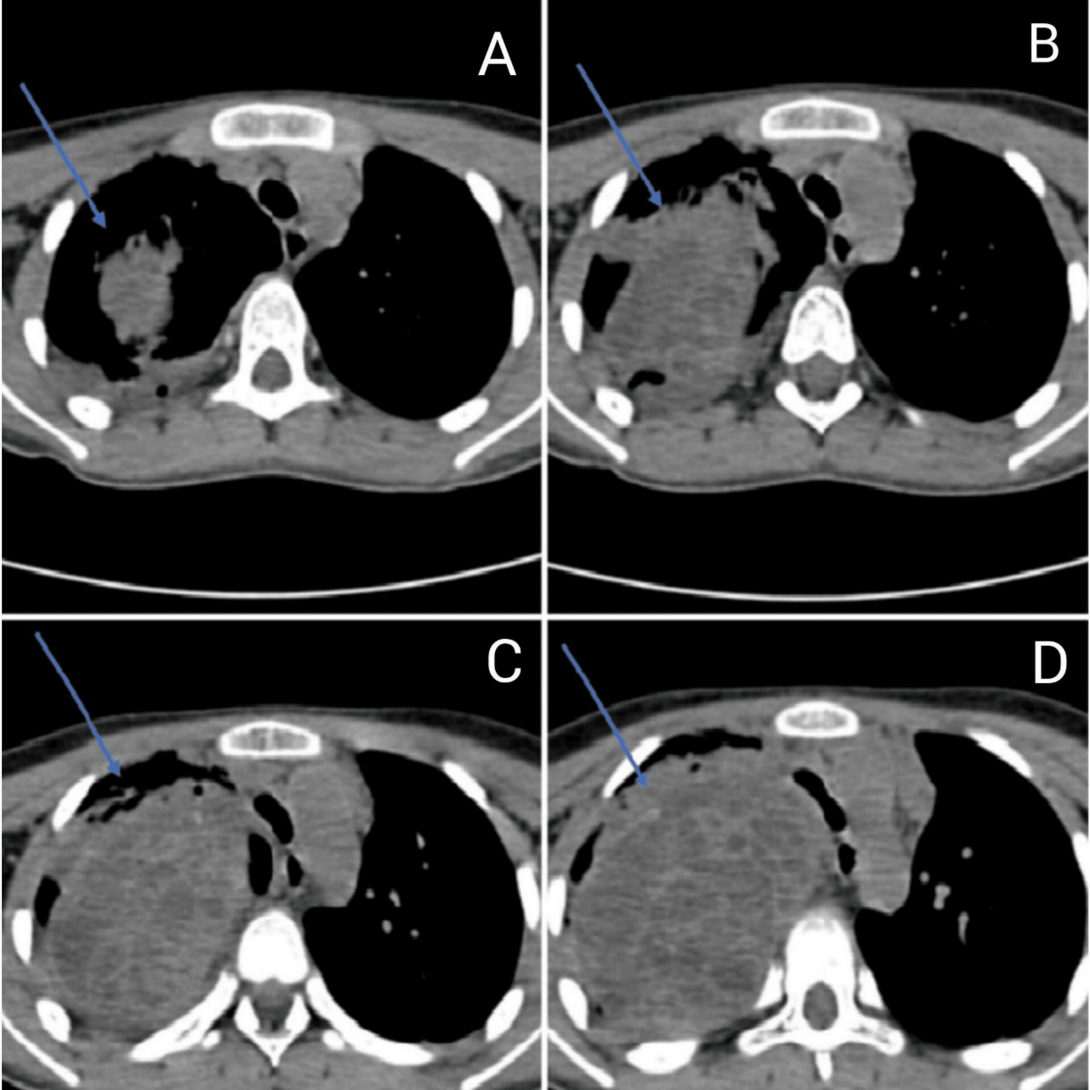
HRCT scan showing mass (blue arrows) at various levels: A) upper part of trachea; B) lower part of trachea; C) just above the carina; D) at the level of carina HRCT, high-resolution computed tomography.

**Figure 3:**
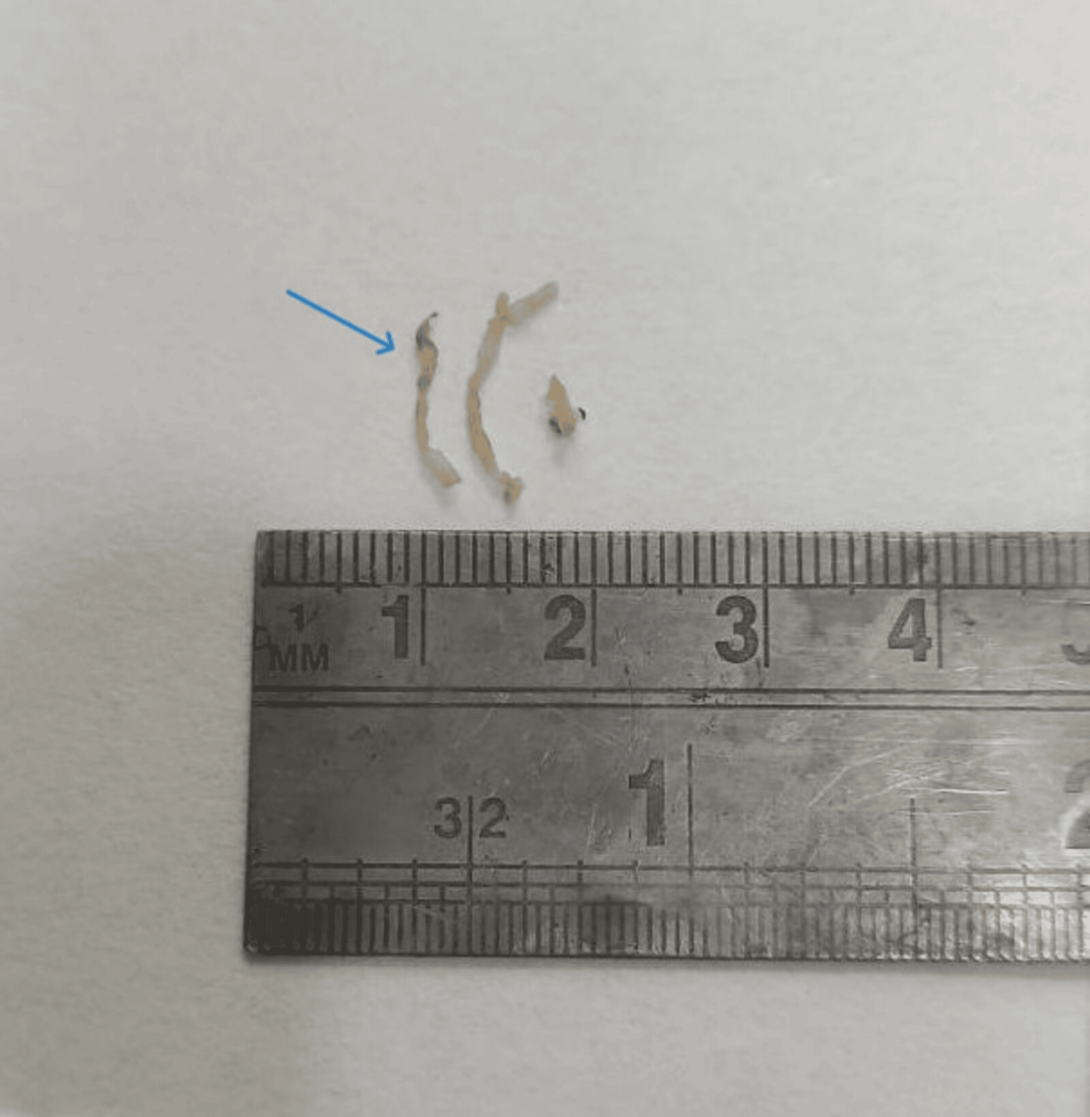
Biopsy specimen of right pleural-based mass lesion (blue arrow)

**Figure 4:**
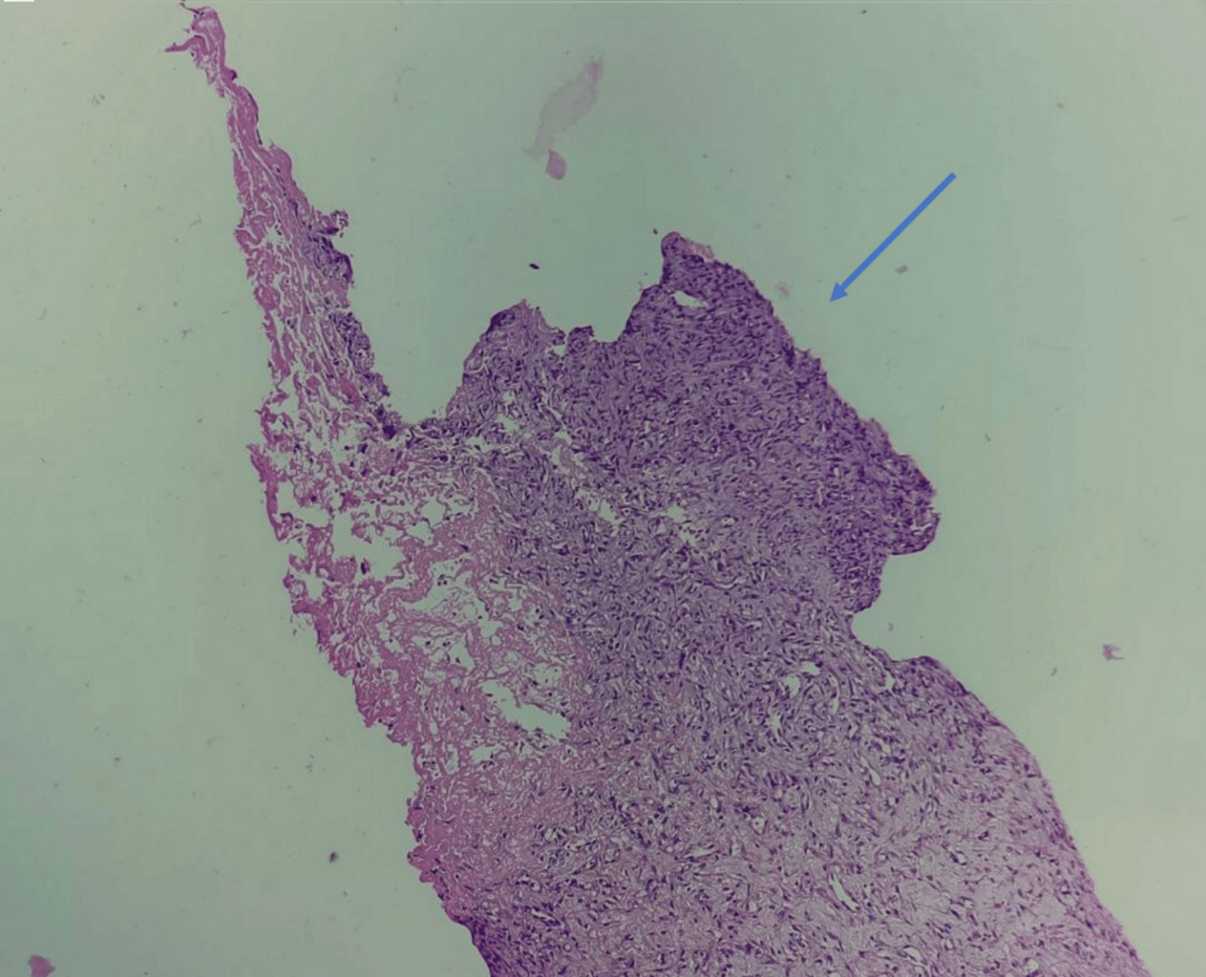
Haematoxylin and eosin-stained section showing spindle-to-oval cells (10× magnification - blue arrow)

**Figure 5:**
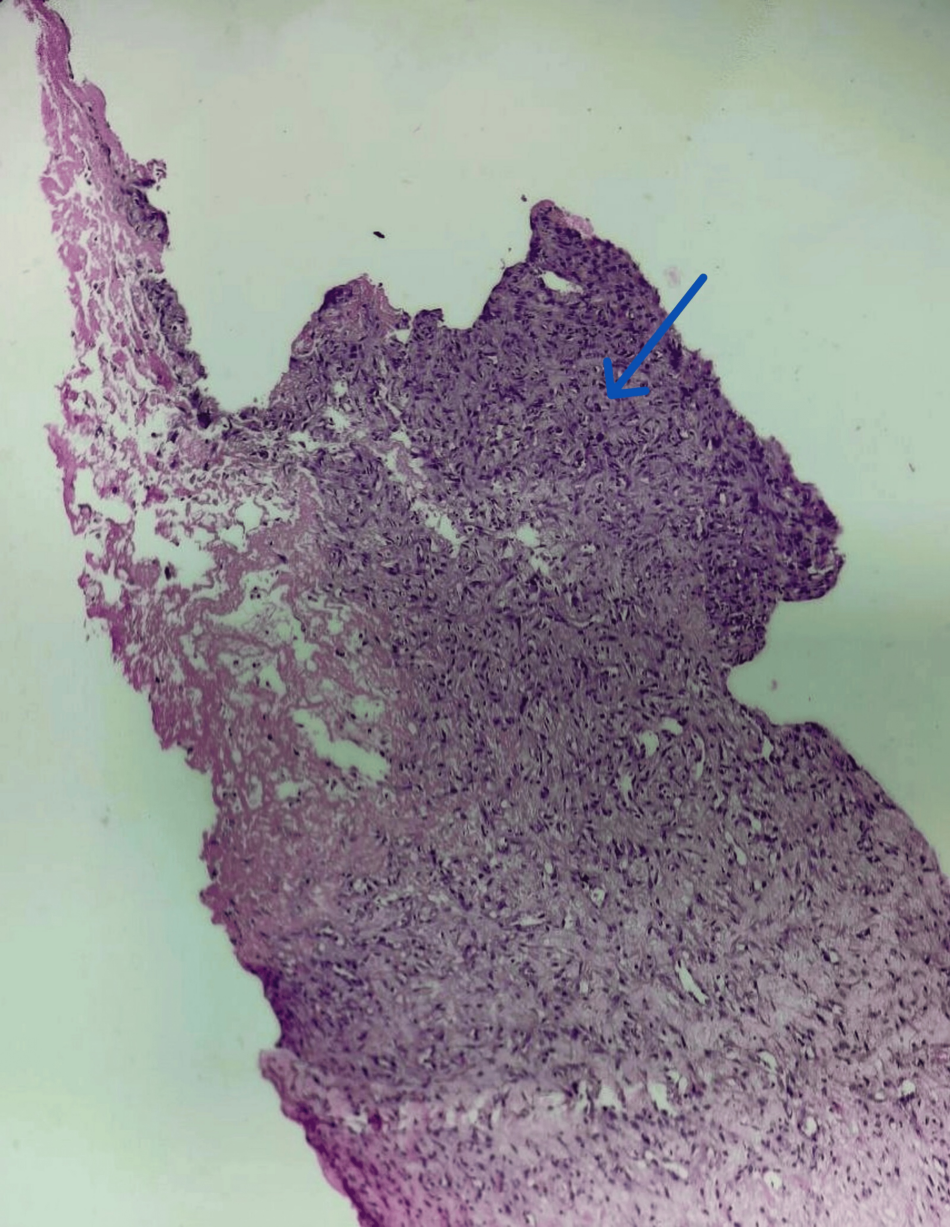
Cells showing neural differentiation (40× magnification, blue arrow) on haematoxylin and eosin stain

**Figure 6:**
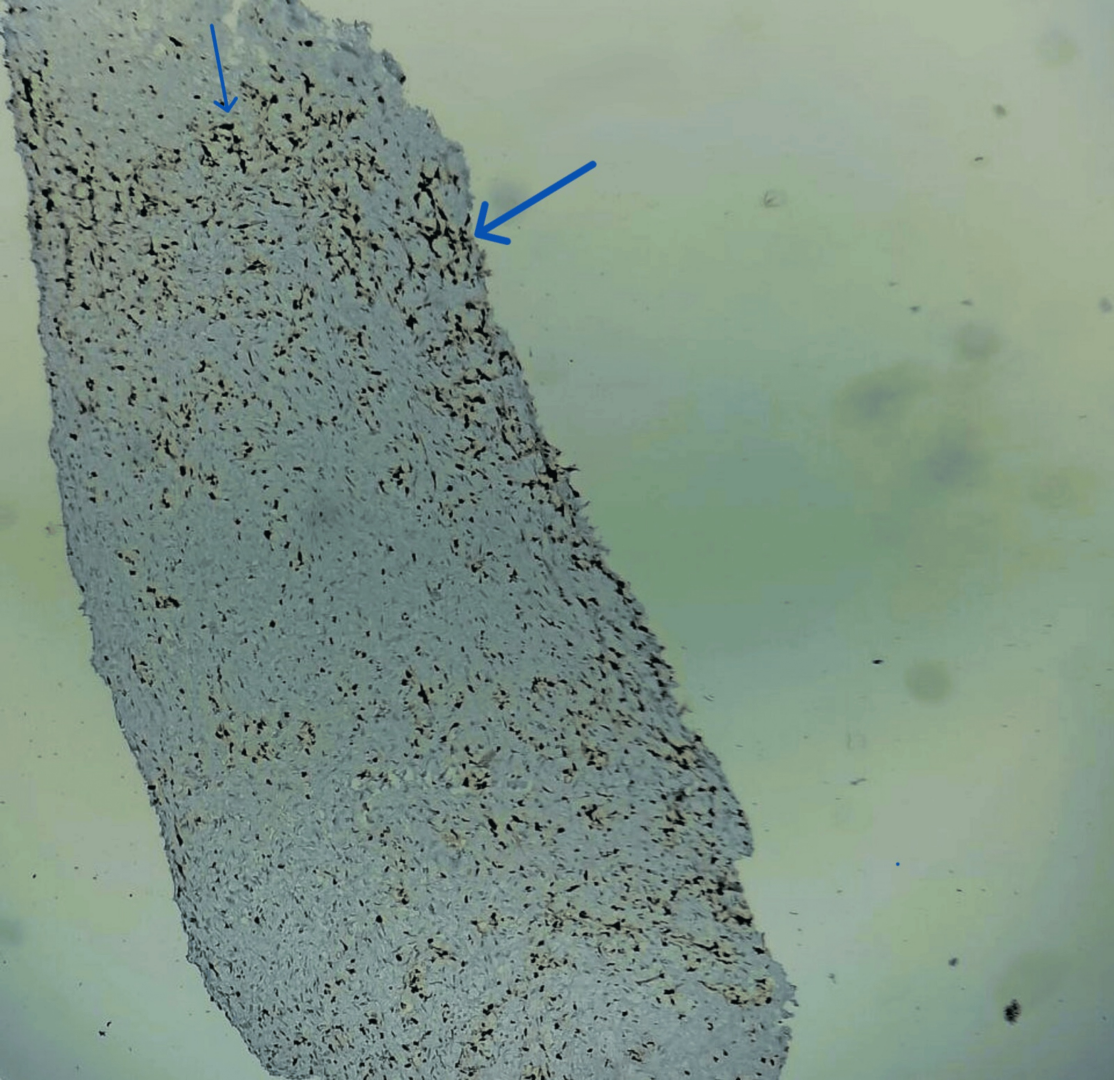
Cells showing immunoreactivity to immunohistochemical marker vimentin (blue arrows)

A 13-year-old girl presented at the emergency department complaining of fever, cold, cough with expectoration, dyspnoea that persisted for 15 days, and generalized weakness that persisted for three months. There was no history of hemoptysis in the past. The patient had previously been admitted to a government hospital in Central India, where investigations revealed elevated total leucocyte counts and pleural effusion. Pleural fluid microscopy also showed a total leucocyte count of approximately 12,000-13,000 cells/cumm with neutrophils 85% and lymphocytes 15%, and ADA (adenosine deaminase) 28.8. The patient received intravenous antibiotic treatment; however, her health did not improve, and she was subsequently transferred to our hospital for more intensive treatment. Upon examination, the patient had a dyspnoeic respiratory rate of 28 breaths per minute, 86 beats per minute pulse, and a blood pressure of 110/70 mm Hg. Upon auscultation, the right side of the chest demonstrated reduced breath sounds. The patient maintained O_2_ at room temperature with nasal prongs at a flow rate of 6 L per minute. Hemoglobin was 11.1 g/dL, total leucocyte count was 13,500 cells/cumm, and platelet count of 4 lakhs/cumm, as per blood investigations. The patient was given intravenous antibiotics and treated accordingly. Furthermore, chest radiography showed a large homogeneous opacity in the right mid and lower zone with cardiophrenic and costophrenic angle obliteration silhouetting right heart border (Figure 1). Additionally, a thoracic HRCT (high-resolution computed tomography) scan revealed a sizable right pleura-based mass lesion with vertebral metastases (Figure 2). An ultrasound-guided biopsy from the mass was done and sent to the histopathology laboratory. Multiple, whitish, thread-like tissue pieces aggregating 1 x 0.5 cm were received in the histopathology lab (Figure 3). Sections stained with haematoxylin and eosin revealed small, mainly spindle-to-oval cells with neural cell differentiation, significant vascularity, and necrosis (Figures 4, 5). In addition, vimentin positivity and a Ki67 score greater than 20% were demonstrated by immunohistochemical staining (Figure 6). Also, the cells showed positivity to epithelial membrane antigen (EMA) and CD99. As per the guidelines for treating peripheral neuroectodermal tumours, chemotherapy was initiated. National Cancer Control Programme Chemotherapy Regimen was followed. The patient was administered etoposide, ifosfamide and mesna (IE therapy). The patient completed five cycles of chemotherapy with a good response to treatment. The patient and her family expressed satisfaction with all of the findings as well as the treatment they received.

## Discussion

Rare malignant tumours called PNETs originate from primitive nerve cells [1]. These are malignant, small, round-cell tumours that are very aggressive. They are frequently referred to as CNS or central PNETs and can manifest as medulloblastomas in the CNS [2]. It is known that children and young adults can develop PNET, an uncommon malignant tumour of the chest wall [3]. Askin described Askin's tumour as a PNET in the thoracopulmonary area [4]. More specifically, it originates from the paravertebral region of the chest wall's soft tissues. Members of the Ewing family of cancers include PNET, Askin's tumour, and Ewing's sarcoma. These tumours are called Askin's tumour when observed in the thoracopulmonary area [5]. PNET of the chest wall frequently manifests as a mass on the chest wall, respiratory difficulty, or chest discomfort [3]. A soft-tissue-density mass in the chest wall is the most typical radiological appearance, and it is occasionally associated with pleural effusion and/or rib erosion [6]. The characteristic CT image seen in patients with Askin's tumours/PNET is a heterogeneous mass of chest wall origin with areas of necrosis and hemorrhage, with or without an intrathoracic component [7]. Tumour growth might compress the lung beneath it or enter it directly. PNET, neuroblastoma, rhabdomyosarcoma, lymphoma, and Ewing's sarcoma are small round cell tumours affecting young adults and children [8]. Both PAS and CD99 are positive on immunohistochemistry in the case of Askin’s tumour. In our instance, the patient had a fever, cough, dyspnoea, and generalized weakness when she presented. HRCT revealed a sizable right pleura-based mass. The biopsy from the mass lesion revealed small, mostly spindle-to-oval cells with neural cell differentiation, significant vascularity, and necrosis in sections stained with haematoxylin and eosin. Vimentin positivity and a Ki67 score greater than 20% were detected by immunohistochemistry. These findings led us to the diagnosis of Askin’s tumour. The established course of treatment for this tumour entails neoadjuvant chemotherapy, surgical tumor removal, and postoperative chemotherapy, either with or without radiation [4].

## Conclusions

Askin’s tumour is a childhood tumour of rare occurrence. Imaging studies and an immunohistochemistry workup are necessary for the diagnosis. This case of Askin's tumour presented a challenging but managed clinical scenario. Through diligent diagnostic evaluation involving imaging studies and histopathological examination, the tumour was accurately identified. Treatment comprised a multimodal approach, incorporating chemotherapy, surgery, and possibly radiation therapy, tailored to the individual patient's needs and tumour characteristics.
